# Mismatch Repair Protein and Microsatellite Instability Analysis in Pancreatic Ductal Adenocarcinoma

**DOI:** 10.3390/jcm15041411

**Published:** 2026-02-11

**Authors:** Ioan Cătălin Bodea, Andra Ciocan, Florin Vasile Zaharie, Radu Vidra, Ștefan Ursu, Răzvan Alexandru Ciocan, Răzvan George Bogdan, Sorana D. Bolboacă, Filip Cristian Tocoian, Bobe Petrushev, Roxana Liana Popa, Nadim Al Hajjar

**Affiliations:** 1 Department of Surgery, “Iuliu Hațieganu” University of Medicine and Pharmacy, Croitorilor Street, No. 19–21, 400162 Cluj-Napoca, Romania; bodea_ioan_catalin@elearn.umfcluj.ro (I.C.B.); zaharie.vasile@umfcluj.ro (F.V.Z.); ursu_stefan@elearn.umfcluj.ro (Ș.U.); tocoian_filip_cristian@elearn.umfcluj.ro (F.C.T.); nadim.alhajjar@umfcluj.ro (N.A.H.); 2 ”Octavian Fodor” Regional Institute of Gastroenterology and Hepatology, Croitorilor Street, No. 19–21, 400162 Cluj-Napoca, Romania; radu.vidra@irgh.ro; 3 MEDFUTURE Biomedical Research Institute, Department of Personalized Medicine and Rare Diseases, “Iuliu Hatieganu” University of Medicine and Pharmacy, 400347 Cluj-Napoca, Romania; 4 Department of Surgery-Practical Abilities, “Iuliu Hațieganu” University of Medicine and Pharmacy, Marinescu Street, No. 23, 400337 Cluj-Napoca, Romania; razvan.ciocan@umfcluj.ro; 5 Department of Plastic Surgery, “Victor Babes” University of Medicine and Pharmacy, 300041 Timisoara, Romania; 6 Department of Medical Informatics and Biostatistics, “Iuliu Hatieganu” University of Medicine and Pharmacy Cluj-Napoca, Louis Pasteur Street, No. 6, 400349 Cluj-Napoca, Romania; sbolboaca@umfcluj.ro; 7 Department of Pathology, “Octavian Fodor” Regional Institute of Gastroenterology and Hepatology, Croitorilor Street No. 19–21, 400162 Cluj-Napoca, Romania

**Keywords:** MSH2, MSH6, MLH1, PMS2, molecular pathway, DNA mutation, targeted therapy, pancreatic ductal adenocarcinoma

## Abstract

**Introduction:** Pancreatic ductal adenocarcinoma (PDAC) represents one of the most aggressive, heterogeneous, and lethal malignancies in humans. Mismatch repair (MMR) proteins constitute a fundamental component of the DNA mismatch repair pathway, which is responsible for correcting replication-associated errors, including incorrect base pairings and small insertions or deletions. This study aims to evaluate the immunohistochemical expression of MSH2, MSH6, MLH1, and PMS2 in resected PDAC and to analyze their association with pTNM stage, perineural and lymphovascular invasion, HER2 and HER3 expression, and tumor volume. **Methods:** A cohort of 106 patients with currative intent Whipple procedure was evaluated, their corresponding paraffin blocks and slides were analyzed using tissue microarray. Immunohistochemical analysis of MLH1, PMS2, MSH2, and MSH6 was performed. Patients were grouped based on MMR expression profiles: isolated MutS loss (MSH2/MSH6), and isolated MutL loss (MLH1/PMS2). **Results:** Among the 106 subjects evaluated, 13 (12.3%) exhibited isolated MutS complex loss and 16 (15.1%) showed MutL complex loss. A total of 7 patients (6.6%) demonstrated concurrent loss of all four MMR proteins, representing a pattern suggestive of MMR deficiency MSI-H. These ones were significantly younger (median 56 vs. 64 years, *p* = 0.0492) and had distinct T-stage distribution (*p* = 0.0237). Two intermediate subgroups were identified: five patients with isolated MutL loss and one patient with isolated MutS loss. HER3 positivity was observed in 3/5 of the intermediate MutL cases and HER2 positivity in only one. **Conclusions:** MMR deficiency and potential MSI-H status were identified to be relevant prognostic biomarkers for pancreatic cancer patients, with MSI-H patients displaying a younger age and distinct tumor features.

## 1. Introduction

Pancreatic cancer (PC), primarily pancreatic ductal adenocarcinoma (PDAC), remains a formidable global health burden. According to last statistics in 2025 there were 510,992 new cases of PC worldwide, with 467,409 deaths, with 67,440 new cases (representing 3.3% of all new cancer cases) reported by NIH (National Cancer Institute) in USA and 51.980 estimated deaths (representing 8.4% of all cancer deaths), with a 5-year relative survival of 13.3%, [1]. Prognosis is highly dependent on the clinical stage at presentation; while localized disease maintains a 5-year survival rate of 44%, this figure precipitously declines to 3% in the presence of distant metastasis [1]. Despite demographic aging, age-standardized rates have modestly declined in the same period, suggesting a complex interplay of risk factor dynamics and healthcare access. In OECD (Organization for Economic Co-operation and Development) countries, pancreatic cancer accounts for around 8% of all cancer-related deaths, highlighting its disproportionate lethality. These numbers illustrate the aggressive nature of PDAC and the urgent need for improved early detection and more effective therapeutic strategies [1,2].

Prognosis and risk of complications (hemorrhage, pancreatic fistula, prolonged gastric stasis), and early recurrence, in PDAC are influenced by a multifactorial interplay of histopathological and molecular characteristics including tumor subtype (intestinal versus pancreatobiliary), lymph node involvement and ratio, peripancreatic extension, perineural and lymphovascular invasion, R0 resections, tumor differentiation (G1, G2 or G3), proliferation index (Ki-67) and p53 or HER2 overexpression, as well as patient-specific and environmental factors such as advanced age, tobacco use, alcohol consumption, dietary patterns, chronic pancreatitis, pancreatic duct diameter, and, in rare cases, genetic predispositions [3,4].

Surgical resection remains the only potentially curative intervention, with prior neoadjuvant treatment or without, depending on local invasion and lymph nodes involvement; however, merely 15–20% of patients present with resectable disease, and even among those achieving R0 resection, prognosis remains poor. Lots of patients came with locally advanced tumor stages, with vascular involvement, being candidates only for palliative treatment [5,6,7]. R0 resection is strongly associated with better overall survival (OS) and disease-free survival (DFS). For example, a large German registry study (~6000 PDAC resections) found median OS was 19.3 months for R0 vs. 13.4 months for R1; R0 was independently protective [8]. Postoperative recurrence rates remain exceptionally high (80–85%), and most patients ultimately succumb to the disease despite microscopic margin clearance [9,10]. In this context, it is imperative to search for the cause of the high recurrence rate of the disease, the histopathological pTNM staging and mechanisms of lymphovascular and perineural invasion having a key role in the local recurrence rate. Adjuvant chemotherapy offers only modest survival benefits, with median overall survival hovering around 12 months, and current standard regimens have yet to yield substantially improved outcomes [11,12]. Consequently, the development of targeted therapies is imperative to improve both overall and disease-free survival.

Recent research has focused on molecularly stratified PDAC subsets, one of which includes mismatch repair-deficient (MMR-D) tumors [13,14]. Mismatch repair proteins constitute a critical genomic maintenance system that corrects replication-associated errors, including base–base mismatches and small insertion/deletion loops. The MMR pathway primarily involves MutS homologs (MSH2, MSH6- primary mismatch sensor) that recognize DNA mismatches and MutL homologs (MLH1, PMS2) that coordinate downstream excision and repair. Following mismatch detection by MSH proteins, recruitment of MLH complexes initiates removal of the aberrant DNA segment, subsequent resynthesis with the correct nucleotides, and final ligation. Structurally, MSH proteins belong to the AAA+ ATPase family, while MLH/PMS proteins have a GHKL (gyrase, HSP90, histidine kinase, MutL) ATP-binding fold [15]. After binding mismatch and ATP, MutS changes conformation, which may recruit MutL. The mobile C-terminal domain (in full-length MutS) allows the protein to switch between dimer and tetramer forms, which may be relevant for its function on DNA. Dysfunction of this repair machinery results in accumulation of mutations, genomic instability, and heightened oncogenic potential. Due to those complex structural changes, abnormal cell replication and tumor proliferation can be accelerated, as demonstrated by the appearance of tumor processes.

Correct identification of the immunohistochemical profile and of receptors that are positive or overexpressed at the membrane level offers the patient the chance for a targeted, correctly individualized treatment with a high response rate, whether it concerns borderline or inoperable tumors. In 2017, the US FDA approved the immune checkpoint inhibitor pembrolizumab for the treatment of solid tumors exhibiting high microsatellite instability (MSI-H) or deficient mismatch repair (dMMR), independent of tumor origin. In cases where immunohistochemistry (IHC) suggests MMR proficiency but clinical or morphological suspicion of dMMR persists, confirmatory MSI testing is recommended [16].

Recent studies have investigated transmembrane protein receptors and the pathological mechanisms underlying abnormal tumor proliferation and tumoral tissue local invasion. In particular, deficiencies in mismatch repair (MMR) proteins, which lead to the accumulation of DNA-level mutations, have been implicated in the pathogenesis of PDAC. This molecular alteration not only contributes to local tumor invasion but also correlates with pathology staging parameters, including vascular, lymphatic, and perineural invasion [17,18]. Because of the urgent need to obtain a complete immunohistochemical profile of PDAC tumors for neo and adjuvant treatment, the aim of this study is to evaluate and underline the MMR function/disfunction in PDAC and the relationship between MMR and HER2+HER3.

## 2. Materials and Methods

### 2.1. Study Design and Cohort

A five-year (2017–2022) retrospective observational analytical cohort study was conducted, including patients with positive histopathological results of PDAC of the head of the pancreas, without neoadjuvant treatment, after curative intent Whipple procedure performed in a high-volume referral centre for pancreatic surgery (Regional Institute of Gastroenterology and Hepatology “Octavian Fodor” Cluj-Napoca, Romania). Although the Whipple procedure is most commonly performed for PDAC, but it is also indicated for a range of other pathologies, including duodenal adenocarcinoma, ampullary carcinoma, distal cholangiocarcinoma, necrotic haemorrhagic pancreatitis, neuroendocrine tumours, pseudo tumoral pancreatitis, which were part of the exclusion criteria.

Among all eligible surgical cases, 106 PDAC resected specimens were selected for in depth analysis. Case selection was based on the quality of paraffin-embedded tissue, with inclusion of specimens demonstrating optimal preservation on haematoxylin–eosin–stained sections and immunohistochemical confirmation of PDAC after surgery.

Inclusion criteria consisted of patients with histopathologically confirmed PDAC, resectable disease at diagnostic, no history of neoadjuvant chemotherapy or immunotherapy and pancreatoduodenectomy performed with curative margins. Exclusion criteria comprised incomplete clinical records, palliative procedures performed before Whipple surgery, prior neoadjuvant treatment and vascular involvement exceeding 180° of the superior mesenteric artery, or splenomesenteric confluent, and other pancreatic pathologies than PDAC.

### 2.2. MSH2, MSH6, MLH1, and PMS2 Analysis

Mismatch repair (MMR) protein expression was assessed in all 106 specimens, specifically targeting MSH2, MSH6, MLH1, and PMS2. For analytical purposes, patients were grouped according to MMR protein expression. MutS complex loss was defined as concurrent loss of MSH2 and MSH6, while MutL complex loss was defined as concurrent loss of MLH1 and PMS2. Tumors were considered positive for a given protein if nuclear staining was preserved in tumor cells. Based on immunohistochemical expression patterns, tumors were classified into the following categories:

- isolated loss of MutS complex proteins (concurrent loss of MSH2 and MSH6)
- isolated loss of MutL complex proteins (concurrent loss of MLH1 and PMS2),
- complete loss of all four MMR proteins, and
- other atypical or discordant expression patterns.

Tumor resection specimens and corresponding immunohistochemistry (IHC) slides are archived for ten years in the institutional pathology laboratory. Each patient contributed 6–29 slides, three independent pathologists selected the slide best representing the tumor for further analysis. Corresponding paraffin blocks were used to obtain tissue cores for tissue microarray (TMA) construction. Using a 2 mm puncher, tumor samples (tumoral tissue) were transferred from the best tissue quality paraffin block to recipient paraffin blocks containing 24 cores each (23 patient samples + 1 control sample on each blade).

Blocks were incubated at 55–58 °C for 15–20 min and left overnight to facilitate paraffin adhesion. Sections were cut and immunoassayed for MSH2, MSH6, MLH1, and PMS2 using the Leica BOND Max system with BOND ZCL 237 kit Leica Biosystems, Deer park, IL, USA (peroxide block, post-primary reagent, polymer, DAB development, hematoxylin counterstaining). Antibodies used were: MSH2 (clone 79H11, 7 mL), MSH6 (clone EP49, 7 mL), PMS2 (clone EP51, 7 mL), and MLH1 (clone ES05, 7 mL). Each slide was independently evaluated twice by three distinct pathologists to ensure diagnosis accuracy. Slides were scored 0 or 1+ depending to the quality of staining for each marker, after examination of all 4 plates for each case, corresponding to 424 (106 cases × 4 plates/case) positive or negative values. Pathologists were aware that samples were from PDAC confirmed patients but were blinded to clinical records to minimize bias.

For biostatistical analysis, the presence or absence of each receptor was assessed, with four determinations quantified per case. Microsatellite instability was not molecularly assessed in this study. All results are based exclusively on immunohistochemical evaluation of MMR protein expression. Accordingly, tumors exhibiting loss of one or more MMR proteins are reported as MMR-deficient immunohistochemical patterns suggestive of microsatellite instability, without molecular confirmation.

### 2.3. Statistical Analysis

Statistical analysis was exploratory and conducted in Jamovi (version 2.6.26). Graphs were generated in Microsoft Excel. Quantitative variables were reported as median and interquartile range and the differences between groups were tested with Mann-Whitney test. Attribute variables were reported as ratios of the number, who met criteria to the number of eligible patients; Fisher’s exact test was used to test differences between groups.

## 3. Results

A total of 106 patients, ranging in age from 36 to 83 years, were included in the analysis. The predominant tumour grades and stages were G2 and T2, respectively. Comparative analysis revealed no statistically significant differences between male and female patients with respect to most clinicopathological characteristics. The only exception was the presence of metastatic disease, which was detected exclusively in three female patients. Notably, no cases of undifferentiated G4 tumours were identified within the investigated cohort. Tumoral burden was comparable between sexes, with a median tumour diameter of 6.5 cm in male patients and 6.8 cm in female patients. Advanced tumour stages were frequently observed in both groups, with T2-stage tumours accounting for more than half of the cases and T3-stage tumours representing over one quarter of the cases in each sex. Evaluation of nodal involvement demonstrated no statistically significant sex-based differences. N1 disease was present in 45.8% of male patients and 53.2% of female patients, while N2 disease was observed in 37.3% of males and 31.9% of females. During the study period, nine patients died as a direct result of cancer-related causes, including six male and three female patients.

### 3.1. Isolated MutS Complex Loss (MSH2/MSH6)

**Figure 1:**
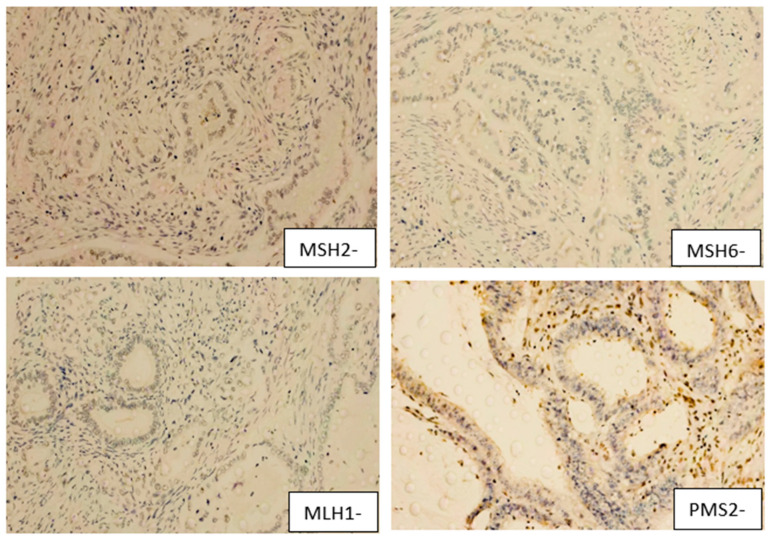
Microscopy images of MSH2, MSH6, MLH1, PMS2 negative status—50× magnification—nuclear signal expression for antibodies MSH2, MSH6, MLH1 and PMS2.

**Figure 2:**
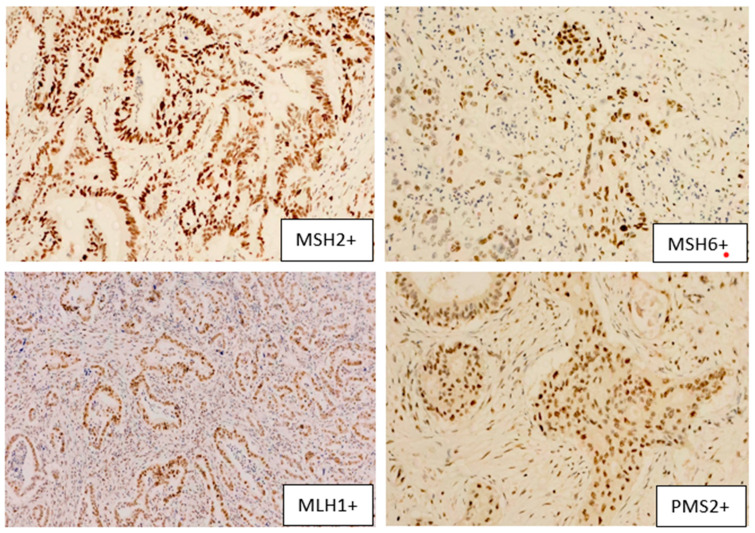
Microscopy images of MSH2, MSH6, MLH1, PMS2 positive status—50× magnification—nuclear signal expression for antibodies MSH2, MSH6, MLH1 and PMS2.

Thirteen patients exhibited concurrent loss of MSH2 and MSH6, consistent with a MutS complex deficiency, suggestive of isolated MutS complex loss (MMR-deficient IHC pattern). Compared to the rest of the cohort, these patients showed similar age, sex distribution, and tumor grade. However, perineural invasion was less frequent in the MutS-deficient group. Lymphatic invasion was observed in 69.2% of the MutS-deficient cases versus 79.5% cases, while vascular invasion was slightly more frequent in the MutS-deficient group (53.8% vs. 43.0%). In both subgroups, G2 differentiation was predominant (Table 1). Regarding HER receptors relationship with MSH, only 2 patients had HER2+ and 3 patients had HER3+. Four examples of MSH2, MSH6, MLH1 and PMS2 are presented below in Figure 1 and Figure 2, in both positive and negative staining, microscopic approach with 50× magnification, photos taken with Zeiss Microscope, Zeiss Instruments SRL, Bucharest, Romania.

### 3.2. Isolated MutL Complex Loss (MLH1/PMS2)

Sixteen patients showed concurrent loss of MLH1 and PMS2, indicating MutL complex deficiency. Subjects had clinicopathological characteristics similar to the rest of the cohort. Nonetheless, lymphatic invasion was observed in 87.5% of the MutL-deficient group versus 76.6% among patients with preserved MutL expression. Vascular invasion occurred in 62.5% of MutL-deficient cases compared to 41.1% in the MutL-positive group, while perineural invasion was recorded in 75.0% versus 73.3%, respectively (Table 2). Only 2 patients with complex loss of MLH1/PMS2 had HER2+ and 5 had HER3+.

### 3.3. Complete Loss of All Four MMR Proteins

A distinct subgroup of seven patients (6.6%) exhibited loss of all four MMR proteins (MSH2, MSH6, MLH1, PMS2), consistent with an MMR-deficient IHC profile. A statistically significant association was found between MutS and MutL negativity (Fisher’s exact test, *p* = 0.0005).

Patients with all negative mismatched were similar to those without all negative mismatched in terms of sex (*p* = 0.4592), tumor differentiation grade (*p* = 0.3368), tumor diameters (DCC: *p* = 0.1819, DLL: *p* = 0.2632, DAP: *p* = 0.1184), involvement of nearby lymph nodes (*p* = 0.8742), presence of distant metastases (*p* > 0.9999), lymphatic (*p* > 0.9999), vascular (*p* = 0.2372) and perineural invasion (*p* = 0.0773), resection margins (*p* = 0.5897), HER2+ (*p* > 0.9999) and HER3+ (*p* = 0.6968).

Statistically significant differences were observed in age (56 years [49.5 to 60.5] vs. 64 [57.5 to 70] years; *p* = 0.0492), and T stage (size and growth of the primary tumor (T1: 0/7, T2: 2/7, T3: 4/7, T4: 1/7 vs. T1: 9.1%, T2: 61.6%, T3: 29.3%, T4: 0.0%; *p* = 0.0237).

**Figure 3:**
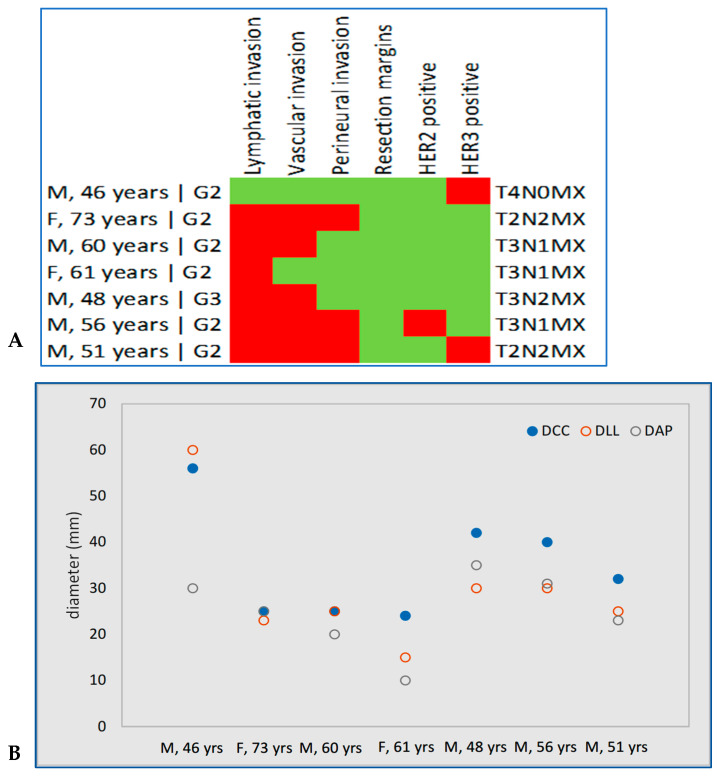
Patients with all four evaluated mismatches: (**A**) characteristics, (**B**) tumor diameters (mm). Red indicates a positive result, and green indicates a negative result.

Six of the seven patients had lymphatic invasion, five had vascular invasion, three had perineural invasion, and all had R0 resection margins. Tumor diameters in this group ranged from 28 to 56 mm craniocaudally, 15 to 60 mm laterolaterally, and 10 to 37 mm anteroposteriorly (Figure 3).

### 3.4. Atypical MMR Immunohistochemical Expression Patterns

Two intermediate subgroups were also identified. The first included five patients who were positive for MSH2 and MSH6, but negative for MLH1 and PMS2, indicating an atypical intermediate profile of microsatellite instability. This group comprised three men and two women. All five had perineural invasion and R0 resection margins. Four patients had G1 or G2 tumors, while one had a G3 tumor. Lymphatic invasion was present in four of the five patients. HER3 positivity was observed in three cases and HER2 positivity in one. Tumor dimensions ranged from 13 to 45 mm craniocaudally, 10 to 30 mm latero-laterally, and 5 to 30 mm anteroposteriorly.

**Figure 4:**
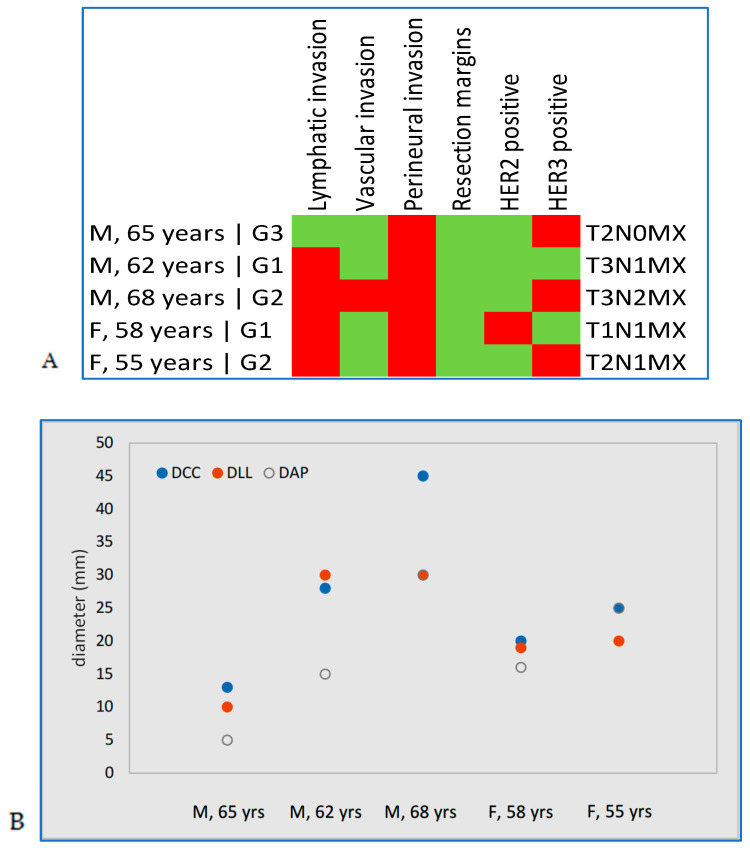
Patients with intermediate microsatellite instability: (**A**) characteristics, (**B**) tumor diameters (mm). Red indicates a positive result, and green indicates a negative result.

The second subgroup consisted of a single male patient, aged 69, with MSH2 and MSH6 loss but MLH1 and PMS2 positivity. This patient had a G1 tumor (T2N0Mx), without any form of invasion or HER2/HER3 expression. Resection margins were clear (R0), and tumor dimensions were 35 mm DCC, 20 mm DLL, and 27 mm DAP (Figure 4).

## 4. Discussion

According to literature, 1.2% of MMR reduced (r-MMR) was found in a cohort of 164 patients with PDAC. Prezioso E. et al. found in their study 0.8% loss of MMR stability, but all of them had also Lynch Syndrome [19]. In our study the percent was 4.71%, higher rate than others, including a study with 418 periampullary adenocarcinoma that finds 3.5% of microsatellite instability [19]. Laghi L. et al. declare 0.3% MSI instability out of 338 PDAC cases [20]. In Humphris J.L et al. study, MLH1 and MSH2 instability was found in 1% from 385 patients with PDAC [21].

In our study, the presence of MutS homologs, which recognize DNA mutations, MSH2 and MSH6 combination was negative in 13 cases (Table 2), most of the cases were part of G2 differentiation grade, without any influence in tumor diameters comparing to normal positive group. When it comes to T stage, 12 cases were T2 and T3 cases, positive lymph nodes in 11 cases. We found lymphatic invasion in 9 cases, vascular and perineural in half cases, but without any statistic significant percent for differentiation.

When it comes to the system which coordinates downstream excision and repair, MutL homologs MLH1 and PMS2 combination, we found 16 cases with negative qualitative value. There is no significant difference between male and female, differentiation grade was mostly G2 with 12/16 cases. Comparing to MSH2 and MSH6 group, tumor diameters was smaller in cases with MLH1 and PMS2. Similar to first group, T stage was mostly T2 and T3. According to pTNM staging we found that lymphatic invasion was positive in 14/16 cases, vascular invasion was positive in 10/16 cases and perineural invasion was positive in 12/16 cases. Comparing the groups, we found that in cases with MLH1 and PMS2 negative, with microsatellite instability, patients had smaller diameters of the tumoral mass, but more aggressive regional invasion. We found no significant association between the negativity of MutL and MutS complex and HER2/HER3 presence on membrane surface. Seven patients in our study were identified with all of those receptors negative (both MutL and MutS). From those 7 patients, 6 of them had lymphatic invasion and 5 had vascular invasion. We didn’t find any specific relationship between those cases and HER2/HER3 presence. Moreover, we found 5 patients with positive MSH2 and MSH6 and negative MLH1 and PMS2. Without any exception, all patients had perineural invasion and 4/5 of them had lymphatic involvement, 3/5 of them had also HER3 positive on membrane surface.

Microsatellite instability (MSI) and mismatch repair (MMR) deficiency represent important, but relatively uncommon molecular features in pancreatic ductal adenocarcinoma (PDAC). In most cases of PDAC, the MMR system—comprising the proteins MLH1, PMS2, MSH2, and MSH6—functions normally, and the tumors are microsatellite stable. However, a small subset of PDACs, typically estimated at 1–2%, exhibit high MSI (MSI-H) due to loss of one or more MMR proteins [18]. Although rare, these MSI-H/MMR-deficient tumors are clinically significant because they may respond favorably to immune checkpoint inhibitors. In PDAC, identifying MSI or MMR deficiency can be challenging, as these tumors may not display the typical histologic features seen in MSI-H colorectal or endometrial cancers. Nevertheless, routine IHC for MLH1, PMS2, MSH2, and MSH6 or molecular MSI testing can reliably determine MMR status. When loss of MMR proteins is detected, further evaluation for Lynch syndrome may be warranted, especially in younger patients or those with relevant family histories. Clinically, MSI-H/MMR-deficient PDAC holds therapeutic relevance because it may respond to PD-1 blockade therapies such as pembrolizumab. Although responses are not as frequent as in other MSI-H cancers, identifying this subset is crucial, as it offers one of the few opportunities for targeted immunotherapy in an otherwise treatment-resistant disease.

Even though the evolution of diagnostic and treatment methods is constantly developing, pancreatic cancer continues to represent a tremendous therapeutic challenge. Epidemiological projections suggest that by 2030, it will constitute the second leading contributor to cancer-associated mortality, second only to lung cancer [22]. The disease’s pronounced biological heterogeneity, deep retroperitoneal location, nonspecific symptomatology, tardive clinic signs appearance and the lack of reliable population-based screening strategies contribute to the fact that the majority of cephalopancreatic neoplasia are identified at advanced, surgically unresectable stages [23]. Therefore, the development of effective neoadjuvant treatment algorithms aimed at tumor downstaging and cytoreduction is essential, ideally incorporating approaches that target the suppression or modulation of overexpressed cellular receptors.

Pembrolizumab, an anti–programmed death-1 (PD-1) immune checkpoint inhibitor, is currently the only immunotherapy approved by the U.S. Food and Drug Administration (FDA) for advanced pancreatic ductal adenocarcinoma (PDAC) exhibiting mismatch repair deficiency (dMMR) or high microsatellite instability (MSI-H). In May 2017, the FDA granted approval for pembrolizumab in patients with unresectable or metastatic MSI-H/dMMR solid tumors who had progressed on prior therapy and had no satisfactory alternative treatment options. This marked the FDA’s first tissue-agnostic approval. The decision was based on pooled data from five multi-cohort, single-arm clinical trials evaluating pembrolizumab in patients with metastatic or unresectable solid tumors, who had received a median of two prior lines of therapy. Among 149 patients with MSI-H/dMMR tumors across these studies, 59 achieved an objective response, corresponding to an overall response rate (ORR) of 36.9%, including a complete response rate of 7% [24,25].

The credibility of this study lies in the utilization of a well-defined cohort with histopathologically confirmed pancreatic ductal adenocarcinoma (PDAC) after Whipple procedure. All patients underwent standardized, curative-intent pancreatoduodenectomy at a high-volume center, ensuring a homogeneous surgical and perioperative approach. Furthermore, stringent inclusion criteria based on histological specimen quality facilitated highly reliable pathological and immunohistochemical profiling.

Study limitations primarily include a high rate of postoperative loss to follow-up, which prevented the collection of six- and twelve-month outcome data, as many patients continued adjuvant treatment in external institutions, without recent data access for present statistic. Furthermore, several patients died within the first three postoperative months, and the specific causes of death—whether attributable to tumor progression, surgical complications, or exacerbation of preexisting comorbidities—could not be determined. Another constraint was the relatively small sample size, as all of those receptors’ analyses are not part of standard immunohistochemical panels and were conducted solely for research purposes.

These findings should be interpreted with caution and may reflect methodological factors, including the use of tissue microarrays with 2-mm cores, potential intratumoral heterogeneity of MMR protein expression, and the absence of molecular MSI confirmation. Precise immunohistochemical characterization of all receptors implicated in dysregulated cellular proliferation and abnormal cell invasion is critical for establishing the morpho pathological profile of each tumor. Considering the well-documented diagnostic and therapeutic relevance of MSI instability in colorectal cancer, as well as supporting evidence from phase I–II trials in gastric and pancreatic cancer, incorporation of MSH2, MSH6, MLH1 and PMS2 evaluation into routine pre- and postoperative histopathological workflows is warranted. Preoperative analysis through endoscopic ultrasound-guided biopsy (EUS) can guide neoadjuvant therapeutic planning, enabling oncologists to tailor targeted chemotherapeutic and immunotherapeutic regimens based on accurate staging and receptor expression patterns. The present study utilized 2-mm core samples from paraffin-embedded blocks, indicating that presence and absence assessment could feasibly be performed on standard Trucut biopsy specimens.

Overall, the study underscores the feasibility and value of routine immunohistochemical assessment of MMR proteins in PDAC. Integration of MMR profiling into pre- and postoperative workflows can inform personalized treatment strategies, including potential eligibility for immunotherapy with PD-1 inhibitors. These findings support the growing evidence that molecular characterization, alongside histopathological evaluation, is essential for optimizing patient management.

## 5. Conclusions

Lymphatic invasion was observed in 69.2% of the MutS-deficient cases versus 79.5% in MMR-positive cases, while vascular invasion was slightly more frequent in the MutS-deficient group (53.8% vs. 43.0%). Consecutively, lymphatic invasion was observed in 87.5% of the MutL-deficient group versus 76.6% in preserved MutL expression. Vascular invasion occurred in 62.5% of MutL-deficient cases compared to 41.1% in the MutL-positive group, while perineural invasion was recorded in 75.0% versus 73.3%, respectively. MSH2, MSH6, MLH1/PMS2 disfunction has little connection with HER2 and HER3 family, and the treatment should be considered distinctively. Ultimately, targeted analysis of MSI and MMR status is able to select the subset of pancreatic cancer patients most likely to benefit from emerging precision oncology approaches.

## Figures and Tables

**Table 1:** Characteristics of the investigated cohort by MSH2 and MSH6 status.

Characteristic	Negative (*n* = 13)	Positive (*n* = 93)	*p*-Value
Age, years ^a^	59 [51 to 69]	64 [57 to 70]	0.2698
Sex ^b^			0.3780
Male	9/13	50/93	
Female	4/13	43/93	
Differentiation Grade ^b^			0.4009
G1	1/13	23/93	
G2	9/13	56/93	
G3	3/13	14/93	
Tumor diameters ^a^			
DCC	25 [24 to 35]	28 [22 to 35]	0.8315
DLL	25 [18 to 30]	24 [18 to 30]	>0.9999
DAP	25 [20 to 30]	20 [15 to 25]	0.1127
pT stage ^b^			0.1293
pT1	0/13	9/93	
pT2	7/13	56/93	
pT3	5/13	28/93	
pT4	1/13	0/93	
pN ^b^			0.9242
0	2/13	15/93	
1	7/13	45/93	
2	4/13	33/93	
M ^b^			>0.9999
1	0/13	3/93	
X	13/13	90/93	
Lymphatic invasion ^b^	9/13	74/93	0.4727
Vascular invasion ^b^	7/13	40/93	0.5561
Perineural invasion ^b^	6/13	72/93	0.0378
Resection margins ^b^			
R0	13/13	78/93	
R1	0/13	15/93	
HER2 positive ^b^	2/13	12/93	0.6808
HER3 positive ^b^	3/13	41/93	0.2300

^a^ data are reported as median [Q1 to Q3], where Q is the value of quartile; Mann-Whitney test was applied to compare the groups; ^b^ data are reported as number (%); Fisher’s exact test was used to compare the groups; G1—well differentiated (low grade), G2—moderately differentiated (intermediate grade), G3—poorly differentiated (high grade), Tumor diameters: DCC—craniocaudal diameter, DAP—anteroposterior diameter, DLL—lateral diameter; TNM classification; Resection margins: R0—free of tumor tissue or R1—with microscopic tumor tissue present.

**Table 2:** Characteristics of the investigated cohort by MLH1 and PMS2 status.

Characteristic	Negative (*n* = 16)	Positive (*n* = 90)	*p*-Value
Age, years ^a^	54.5 [59 to 62.75]	58 [64.5 to 71]	0.5406
Sex ^b^			>0.9999
Male	9/16	50/90	
Female	7/16	40/90	
Differentiation Grade ^b^			0.5523
G1	2/16	22/90	
G2	12/16	53/90	
G3	2/16	15/90	
Tumor diameters ^a^			
DCC	23 [26 to 34]	23 [28 to 35]	0.8246
DLL	20 [25 to 30]	18 [24 to 30]	0.3969
DAP	15.75 [20 to 26.25]	15.25 [20 to 25]	0.8236
pT stage ^b^			0.1030
T1	1/16	8/90	
T2	7/16	56/90	
T3	7/16	26/90	
T4	1/16	0/90	
pN ^b^			0.7585
0	2/16	15/90	
1	7/16	45/90	
2	7/16	30/90	
M ^b^			0.3910
1	1/16	2/90	
X	15/16	88/90	
Lymphatic invasion ^b^	14/16	69/90	0.5134
Vascular invasion ^b^	10/16	37/90	0.1712
Perineural invasion ^b^	12/16	66/90	>0.9999
Resection margins ^b^			0.1194
R0	16/16	75/90	
R1	0/16	15/90	
HER2 positive ^b^	2/16	12/90	>0.9999
HER3 positive ^b^	5/16	39/90	0.4209

^a^ data are reported as median [Q1 to Q3], where Q is the value of quartile; Mann-Whitney test was applied to compare the groups; ^b^ data are reported as number (%); Fisher’s exact test was used to compare the groups; G1—well differentiated (low grade), G2—moderately differentiated (intermediate grade), G3—poorly differentiated (high grade), Tumor diameters: DCC—craniocaudal diameter, DAP—anteroposterior diameter, DLL—lateral diameter; TNM classification; Resection margins: R0—free of tumor tissue or R1—with microscopic tumor tissue present.

## Data Availability

Full data available on request from the corresponding author.

## References

[1] Li X., Zhang Y., Yan Z., Jiang W., Rui S. (2025). Global, regional, and national burden of pancreatic cancer and its attributable risk factors from 2019 to 2021, with projection to 2044. Front. Oncol..

[2] Du R., Wang Y., Pan M., Zhu J., Zhao Y., Zhang C., Liu C., Gao Y. (2025). Global, regional, and national burdens of pancreatic cancer attributable to smoking from 1990 to 2021 and the projections to 2035: A systematic analysis from the global burden of disease study 2021. Front. Oncol..

[3] Lowenfels A.B., Maisonneuve P. (2006). Epidemiology and risk factors for pancreatic cancer. Best Pract. Res. Clin. Gastroenterol..

[4] Demirci N.S., Cavdar E., Ozdemir N.Y., Yuksel S., Iriagac Y., Erdem G.U., Odabas H., Hacibekiroglu I., Karaagac M., Ucar M. (2004). Clinicopathologic Analysis and Prognostic Factors for Survival in Patients with Operable Ampullary Carcinoma: A Multi-Institutional Retrospective Experience. Medicina.

[5] Beuran M., Negoi I., Paun S., Ion A.D., Bleotu C., Negoi R.I., Hostiuc S. (2015). The epithelial to mesenchymal transition in pancreatic cancer: A systematic review. Pancreatology.

[6] Hezel A.F., Kimmelman A.C., Stanger B.Z., Bardeesy N., Depinho R.A. (2006). Genetics and biology of pancreatic ductal ad-enocarcinoma. Genes. Dev..

[7] Von Fristsch L., Duhn J., Abdalla T.S.A., Honselmann K.C., Bolm L., Braun R., Kist M., Lapshyn H., Zeissig S.R., Klinkhammer-Schalke M. (2025). An R0 resection margin does improve overall survival after PDAC resection- real-world evidence from 6.000 cases from the German Cancer Registry Group. Eur. J. Surg. Oncol..

[8] Garcea G., Neal C.P., Pattenden C.J., Steward W.P., Berry D.P. (2005). Molecular prognostic markers in pancreatic cancer: A systematic review. Eur. J. Cancer.

[9] Groot V.P., Rezaee N., Wu W., Cameron J.L., Fishman E.K., Hruban R.H., Weiss M.J., Zheng L., Wolfgang C.L., He J. (2018). Patterns, Timing, and Predictors of Recurrence Following Pancreatectomy for Pancreatic Ductal Adenocarcinoma. Ann. Surg..

[10] Allen P.J., Kuk D., Castillo C.F., Basturk O., Wolfgang C.L., Cameron J.L., Lillemoe K.D., Ferrone C.R., Morales-Oyarvide V., He J. (2017). Multi-institutional Validation Study of the American Joint Commission on Cancer (8th Edition) Changes for T and N Staging in Patients with Pancreatic Adenocarcinoma. Ann. Surg..

[11] Neoptolemos J.P., Stocken D.D., Bassi C., Ghaneh P., Cunningham D., Goldstein D., Padbury R., Moore M.J., Gallinger S., Mariette C. (2010). Adjuvant chemotherapy with fluorouracil plus folinic acid vs. gemcitabine following pancreatic cancer resection: A randomized controlled trial. JAMA.

[12] Ramanathan R.K., Belani C.P., Singh D.A., Tanaka M., Lenz H.J., Yen Y., Kindler H.L., Iqbal S., Longmate J., Mack P.C. (2009). A phase II study of lapatinib in patients with advanced biliary tree and hepatocellular cancer. Cancer Chemother. Pharmacol..

[13] Connor A.A., Denroche R.E., Jang G.H., Timms L., Kalimuthu S.N., Selander I., McPherson T., Wilson G.W., Chan-Seng-Yue M.A., Borozan I. (2017). Association of Distinct Mutational Signatures with Correlates of Increased Immune Activity in Pancreatic Ductal Adenocarcinoma. JAMA Oncol..

[14] Bailey P., Chang D.K., Nones K., Johns A.L., Patch A.M., Gingras M.C., Miller D.K., Christ A.N., Bruxner T.J.C., Quinn M.C. (2016). Genomic analyses identify molecular subtypes of pancreatic cancer. Nature.

[15] Fishel R. (2015). Mismatch Repair. J. Biol. Chem..

[16] Le D.T., Durham J.N., Smith K.N., Wang H., Bartlett B.R., Aulakh L.K., Lu S., Kemberling H., Wilt C., Luber B.S. (2017). Mismatch repair deficiency predicts response of solid tumors to PD-1 blockade. Science.

[17] Hu Z.I., Shia J., Stadler Z.K., Varghese A.M., Capanu M., Salo-Mullen E., Lowery M.A., Diaz L.A., Mandelker D., Yu K.H. (2018). Evaluating Mismatch Repair Deficiency in Pancreatic Adenocarcinoma: Challenges and Recommendations. Clin. Cancer Res..

[18] Taieb J., Sayah L., Heinrich K., Kunzmann V., Boileve A., Cirkel G., Lonardi S., Chibaudel B., Turpin A., Beller T. (2023). Efficacy of immune checkpoint inhibitors in microsatellite unstable/mismatch repair-deficient advanced pancreatic adenocarcinoma: An AGEO European Cohort. Eur. J. Cancer.

[19] Prezioso E., Mancheski E., Shivok K., Kaplan Z., Bowne W., Jain A., Lavu H., Yeo C.J., Nevler A. (2024). Assessing Influence of Mismatch Repair Mutations on Survival in Patients After Resection of Pancreatic Ductal and Periampullary Adenocarcinoma. J. Clin. Med..

[20] Laghi L., Beghelli S., Spinelli A., Bianchi P., Basso G., Di Caro G., Brecht A., Celesti G., Turri G., Bersani S. (2012). Irrelevance of Microsatellite Instability in the Epidemiology of Sporadic Pancreatic Ductal Adenocarcinoma. PLoS ONE.

[21] Humphris J.L., Patch A.M., Nones K., Bailey P.J., Johns A., McKay S., Chang D.K., Miller D.K., Pajic M., Kassahn K.S. (2017). Hypermutation In Pancreatic Cancer. Gastroenterology.

[22] Rahib L., Smith B.D., Aizenberg R., Rosenzweig A.B., Fleshman J.M., Matrisian L.M. (2014). Projecting cancer incidence and deaths to 2030: The unexpected burden of thyroid, liver, and pancreas cancers in the United States. Cancer Res..

[23] Adel N. (2019). Current treatment landscape and emerging therapies for pancreatic cancer. Am. J. Manag. Care.

[24] Marcus L., Lemery S.J., Keegan P., Pazdur R. (2019). FDA Approval Summary: Pembrolizumab for the Treatment of Microsatellite Instability-High Solid Tumors. Clin. Cancer Res..

[25] Viale G., Trapani D., Curigliano G. (2017). Mismatch Repair Deficiency as a Predictive Biomarker for Immunotherapy Efficacy. Biomed. Res. Int..

